# Comparative genomics and metabolomics analyses of the adaptation mechanism in *Ketogulonicigenium vulgare*-*Bacillus thuringiensis* consortium

**DOI:** 10.1038/srep46759

**Published:** 2017-04-25

**Authors:** Nan Jia, Ming-Zhu Ding, Yang Zou, Feng Gao, Ying-Jin Yuan

**Affiliations:** 1 Key Laboratory of Systems Bioengineering (Ministry of Education), School of Chemical Engineering and Technology, Tianjin University, Tianjin, 300072, PR China; 2 SynBio Research Platform, Collaborative Innovation Centre of Chemical Science and Engineering (Tianjin), School of Chemical Engineering and Technology, Tianjin University, Tianjin, 300072, PR China; 3 Department of Physics, Tianjin University, Tianjin, 300072, PR China

## Abstract

Adaptive evolution by serial subcultivation of co-cultured *Bacillus thuringiensis* and *Ketogulonicigenium vulgare* significantly enhanced the productivity of 2-keto-L-gulonic acid in two-step vitamin C production. The adaptation mechanism in *K. vulgare*-*B. thuringiensis* consortium was investigated in this study based on comparative genomics and metabolomics studies. It was found that the growth, anti-oxidation, transcription and regulation were significantly enhanced in the adapted consortium. The mutation of the genes, which encode amidohydrolase in adapted *K. vulgare* (K150) and amino acid permease in adapted *B. thuringiensis* (B150), resulted in the increase of some amino acids levels in each species, and further enhanced the metabolic exchange and growth ability of the two species. Besides, the mutation of the gene encoding spore germination protein enhanced the metabolic levels of tricarboxylic acid cycle, and decreased the sporulation in B150, which induced its growth. The mutation of the genes, which encode NADPH nitroreductase in K150 and NADPH-dependent FMN reductase in B150, may enhance the ability of anti-oxidation. Overall, the long-term adaptation of *K. vulgare* and *B. thuringiensis* influenced the global regulation and made them more inseparable in metabolite exchange. Our work will provide ideas for the molecular design and optimization in microbial consortium.

Microbial consortia have become more and more important for industrial application with the development of synthetic biology^1^. Bacterial populations are thought to facilitate the evolution of cooperative behavior through nature selection^2^. For example, *Pseudomonas putida* depends on the partner organism, *Acinetobacter* sp. strain C6, to grow on benzyl alcohol^3^. When the two species are cultured together as biofilms on benzyl alcohol, *P. putida* mutants have an increased ability to attach to *Acinetobacter* sp. strain C6 cells, which leads to a greater overall growth yield in the co-culture biofilm^4^. Besides, many bacteria are accompanied and even outnumbered by their viruses^5^. During the long term evolution, the bacterial hosts of bacteriophages can lower immune barriers to phage infection, thereby facilitating infection by beneficial phages^6^.

The *K. vulgare*-*Bacillus* consortia are widely used in the industrial production of vitamin C^7^. *K. vulgare* was responsible for the conversion of sorbose to 2-keto-L-gulonic acid (2-KGA, the precursor of vitamin C). The *Bacillus* species were used to stimulate the growth of *K. vulgare*^8^. Adaptive evolution is an efficient approach to enhance growth^9^ and environment adaptation^10^. A serial subcultivation-based experimental adaptation (over 150 days) was conducted on the co-cultured *K. vulgare*-*B. thuringiensis* in our previous research, enabling an increased yield of 2-KGA^11^. The significant changes of metabolic interaction^12^ and protein expression^13^ occurred, thus enhancing synergistic cooperation between *K. vulgare* and *B. thuringiensis*. However, the adaptation mechanism of *K. vulgare*-*B. thuringiensis* consortium is still unclear at the genetic level.

In our previous studies, the genome analysis of *K. vulgare*^14^ and *Bacillus* species^15^,^16^ identified their genetic characteristics. Besides, metabolomics approach has been demonstrated on their metabolic exchanges and interactions^17^. A genome study associated with metabolite profiles can reveal the connection between the single nucleotide polymorphisms (SNPs) and metabolism^18^,^19^. In this study, the adaptation mechanism in *K. vulgare*-*B. thuringiensis* consortium was investigated based on comparative genomics and metabolomics studies. Genetic variants of the genes that associate with metabolite modification in the key nucleosides, carbohydrates, or amino acids were studied, which enabled us to further understand the cooperative mechanism and facilitate the optimization of microbial consortium.

## Results

### Comparative genomic and metabolic analysis of the adapted species

**Figure 1:**
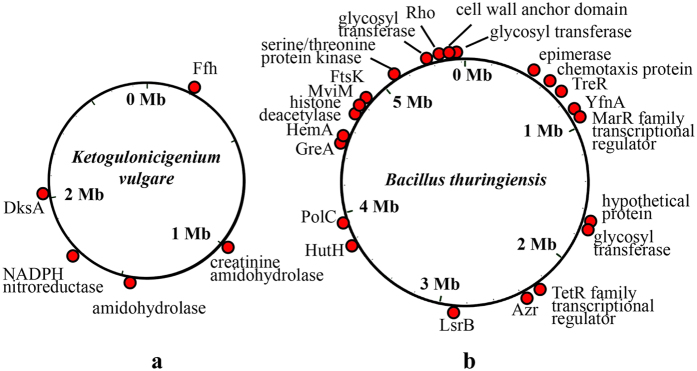
The mutation sites in the adapted species. (**a**) the mutation sites in K150 compared with K0; (**b**) the mutation sites in B150 compared with B0. Schematic representation of the experimental genetic adaptation based on comparative genomic study.

After 150 serial subcultivation of the co-cultured *K. vulgare* and *B. thuringiensis*, the adapted *K. vulgare* (K150) showed higher growth rate and yield of 2-KGA, from 77% to 93%. The complete genome sequence of *K. vulgare* (K0) was obtained in the previous study^14^. To elucidate the mutation site during the experimental evolution process, K150 was resequenced using Illumina technology (Table 1 and Fig. 1a). We found 11 SNPs, including 2 synonymous mutations, 5 non-synonymous mutations and 4 intergenic mutations. The 5 non-synonymous mutations related to the genes encoding signal recognition particle protein (SRP), two amidohydrolases, NADPH nitroreductase and RNA polymerase-binding protein DksA, respectively. The complete genome sequence of *B. thuringiensis* (B0) was also obtained in the previous study^16^ and the adapted *B. thuringiensis* (B150) was resequenced using Illumina technology. We totally found 23 non-synonymous mutations and 12 shifts (Table 1 and Fig. 1b). The 23 non-synonymous mutated genes encoded MarR/TetR family transcriptional regulator, chemotaxis protein, virulence factor, cell division protein, collagen-binding protein, amino acid permease, glycosyltransferase, NADPH-dependent FMN reductase, and so on. The 12 shifts related to the genes encoding spore germination protein, two-component sensor histidine kinase, motility repressor, uracil-DNA glycosylase, molecular chaperone, S-adenosylhomocysteine nucleosidase and some ribosomal proteins, which would make a great change in the gene function.

**Figure 2:**
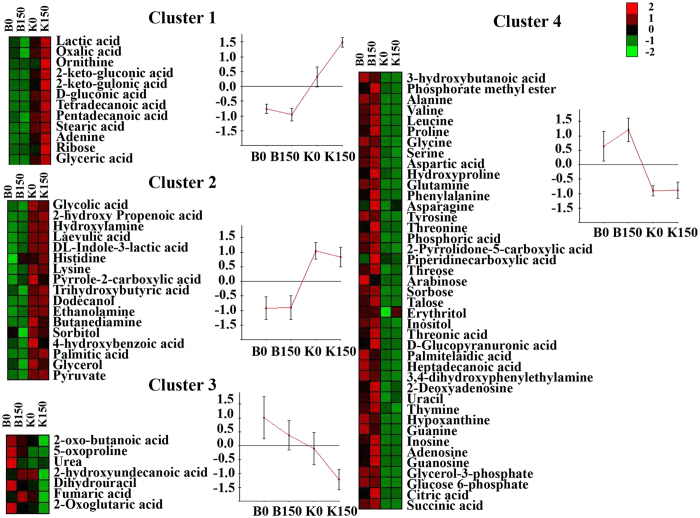
Statistics analysis of the metabolite distribution in the cells. Multivariate data analysis was preformed by hierarchical cluster analysis (HCA) to view the relative differences in the metabolites concentrations among diverse conditions. 77 metabolites were identified and categorized into 4 clusters by the K-means algorithm using Expander 4.1.

The intracellular metabolites of mono-cultured B0, B150, K0 and K150 were analyzed by GC-TOF/MS. 77 metabolites were identified and categorized into 4 clusters by the K-means algorithm using Expander 4.1 (Fig. 2). We found that the levels of 29 metabolites were lower in *B. thuringiensis* than those in *K. vulgare* (clusters 1 and 2). Owing to the defect of amino acids metabolism and glycolysis^14^, the contents of the metabolites related to amino acids and tricarboxylic acid cycle were low in *K. vulgare* (clusters 3 and 4).

### The changes of transcriptional and regulatory capability in the adapted *K. vulgare*-*B. thuringiensis* consortium

Smooth changes in transcriptional and regulatory levels may have significant global response^20^. Two species both have the genetic mutations in transcriptional or regulatory genes, such as the genes encoding RNA polymerase-binding protein DksA and signal recognition particle (SRP) in K150 (Fig. 1a), MarR/TetR family transcriptional regulator, transcription termination factor Rho, transcription elongation factor GreA, two-component sensor histidine kinase and ribosomal proteins in B150 (Fig. 1b). The complex of SRP contains a small molecule RNA and six proteins, which can recognize the signal of peptide terminal on ribosome, combine and stop the peptide synthesis^21^. The mutation of SRP in K150 may influence its signal recognition, elongation arrest and translocation promotion^22^. RNA polymerase can recognize the DNA or RNA strand as a template, which catalyzes nucleoside-5′-triphosphate for RNA synthesis^23^. The mutation of RNA polymerase-binding protein DksA may influence the transcription in *K. vulgare*. By culturing the two species orthogonally on agar plates, the swarming distance of *B. thuringiensis* along the trace of *K. vulgare* on the plate decreased after 150 days’ subcultivation^12^. The mutation of chemotaxis protein and motility repressor MogR may directly influence the swarming ability of *B. thuringiensis* at the genetic level.

### The enhancement of the growth ability of the adapted species in *K. vulgare*-*B. thuringiensis* consortium

K150 acquired higher OD_600_ than K0, and obtained a higher yield of 2-KGA by the serial subcultivation. Our previous studies^13^ identified that K150 acquired more capability compared with K0 in protein degradation. Amidohydrolase (or amidase) is a type of hydrolase that acts upon amide bonds. The mutation of amidohydrolase may influence the amino acids and fatty acids metabolism^24^. After 150 days’ serial subcultivation of the co-cultured *K. vulgare* and *B. thuringiensis*, B150 decreased sporulation, assimilated amino acids more efficiently and had a faster growth rate than the original *B. thuringiensis*^11^. The improvement of the growth state of B150 may connect with diverse mutations, including spore germination protein, and cell division protein FtsK. Besides, three mutated proteins belong to glycosyl transferase family, which catalyze the transfer of saccharide moieties from an activated nucleotide sugar to a nucleophilic glycosyl acceptor (carbohydrate, glycoside, oligosaccharide, or polysaccharide)^25^. The amino acids exchange between *K. vulgare* and *B. thuringiensis* is so inseparable, and five mutations in B150 connect with amino acids metabolism, including amino acid permease, serine/threonine protein kinase, histone deacetylase, glutamyl-tRNA reductase and histidine ammonialyase.

**Figure 3:**
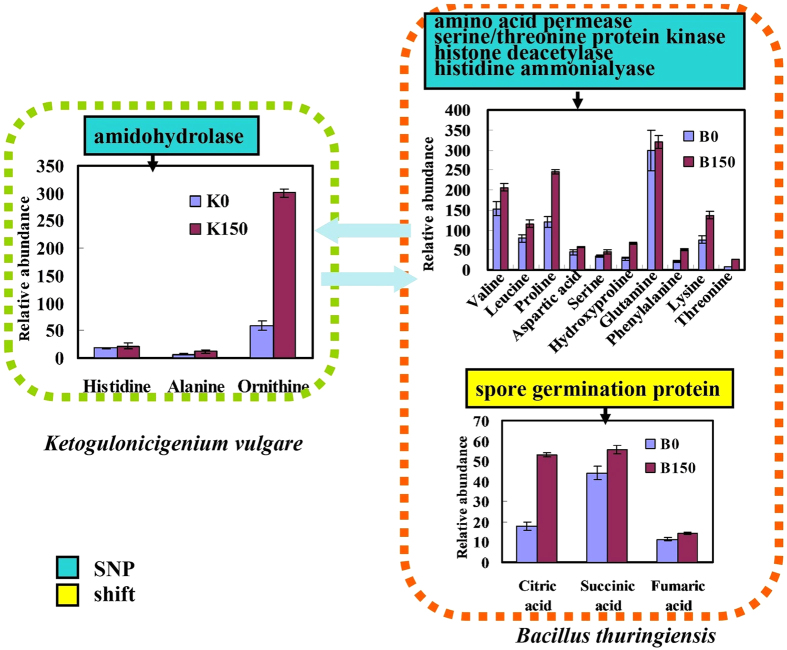
Changes of intracellular metabolites in amino acids metabolism in *K. vulgare*-*B. thuringiensis* consortium. The mutation of amidohydrolase in K150 and amino acid permease in B150 influence the amino acids metabolism, and tricarboxylic acid cycle in B150 was improved significantly. The y-axis was the relative abundance, being calculated by normalization of peak area of each metabolite to internal standard and dry weight of cells. Each value represented mean value of four replicates, and the error bars showed the standard deviations.

The contents of many amino acids increased in B150, including valine, leucine, proline, aspartic acid, serine, hydroxyproline, glutamine, phenylalanine, lysine and threonine. In K150, the contents of histidine, alanine and ornithine increased significantly. Occasionally, the levels of different amino acids increased in the two species, which may due to the long-term cooperative adaptation. Particularity, the content of ornithine was up to 5 fold in K150 than in K0, and the contents of proline, lysine, and threonine increased by 202%, 183%, 335% in B150 than B0 (Fig. 3). These amino acids may be critical to *K. vulgare*-*B. thuringiensis* consortium. There was no significant change in the central carbon metabolism of K150, which may be due to its defect in the related pathway. The tricarboxylic acid cycle in B150 was improved significantly, and the contents of citric acid, succinic acid, fumaric acid were enhanced by 293%, 126%, 125% than those in B0. The advancement of *B. thuringiensis* is conducive to delay its sporulation and plays an important role in the association with *K. vulgare.* The biosynthesis pathway of lipid acid may be also improved. In the purine and nucleoside metabolism, the contents of adenine, inosine, adenosine and uracil increased in both K150 and B150. Leduc *et al*. found that adenine could enhance the growth of *K. vulgare* LMP P-20356 significantly under L-sorbose/CSL medium, and the purine nucleotides biosynthesis pathways in *K. vulgare* were probably insufficient^26^. The improvement of the purine and nucleoside metabolism plays a very important role in the energy and coenzyme supply, thus enhancing the growth ability of the adapted species in *K. vulgare*-*B. thuringiensis* consortium.

### The enhancement of oxidation ability of the adapted species in *K. vulgare*-*B. thuringiensis* consortium

**Figure 4:**
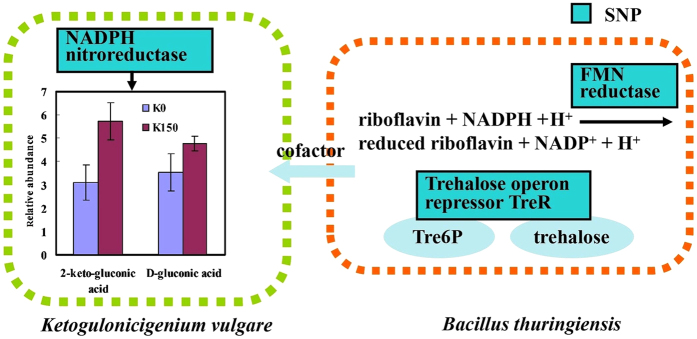
Enhanced oxidation ability of the adapted species in *K. vulgare*-*B. thuringiensis* consortium. NADPH nitroreductase may enhance the oxidation capacity of *K. vulgare* and the contents of 2-keto-gluconic acid, 2-keto-L-gulonic acid and D-gluconic acid in *K. vulgare*, which was further enhanced during long-term adaptation. The mutation of NADPH-dependent FMN reductase and trehalose operon repressor TreR in B150 may affect the supply of flavin and trehalose, thus taking part in the anti-oxidation.

During the ROS defense processes, NADPH provides the reducing power for detoxication and antioxidant^27^. Due to the poor PPP metabolism of *K. vulgare*, the production of NADPH may be insufficient for growth and metabolism. Long-term co-cultured with *Bacillus* species may induce the mutations of the oxidation related genes in *K. vulgare*^28^. The mutation of NADPH nitroreductase may enhance the oxidation capacity of *K. vulgare* from the fermentation environment. The contents of some organic acids (e.g. 2-keto-gluconic acid, 2-keto-L-gulonic acid, D-gluconic acid, tetradecanoic acid, pentadecanoic acid, palmitic acid and stearic acid) in *K. vulgare* were much higher than those in *B. thuringiensis*, and were further enhanced during long-term adaptation (Fig. 4). It indicated that the oxidative ability of *K. vulgare* increased by the improvement of related dehydrogenase expression. Besides, we found two mutated genes related to antioxidant in B150. NADPH-dependent FMN reductase utilizes NADPH as co-factor to convert flavin, and is involved in a number of biological processes that require reduced flavin for their functions^29^. Trehalose operon repressor TreR is capable of binding both the inducer Tre6P and trehalose. The repression activity of TreR is regulated by the ratio of trehalose to Tre6P^30^. These mutations of the genes related to antioxidant in B150 may affect the supply of flavin and trehalose, thus taking part in the anti-oxidation^31^,^32^.

## Discussion

As a tool for adaptive evolution, experimental serial subcultivation of bacteria has been used to study the recovery of species with gene knockouts in central metabolism^33^. Laboratory adaptive evolution followed by genomic sequencing provides the relationship of genotype and phenotype in bacteria^34^. In the present study, the adaptation mechanism in *K. vulgare*-*B. thuringiensis* consortium was investigated based on comparative genomics and metabolomics studies, including the changes of transcriptional and regulatory capability, growth ability and antioxidant ability. The variation of amidohydrolase may significantly influence the metabolite phenotypes and growth state in *K. vulgare*. The contents of histidine, alanine, ornithine, adenine, inosine and adenosine increased significantly. Besides, five mutations in B150 connect with amino acids metabolism and the levels of many amino acids increased significantly. Long-term adaptation of *K. vulgare* and *B. thuringiensis* enhances the ability of amino acids metabolism and makes more inseparable in amino acids exchange. Besides, B150 decreased sporulation due to the mutation of spore germination protein, and had a faster growth rate than the original *B. thuringiensis*. Particularly, the tricarboxylic acid cycle in B150 was improved significantly. The inhibition of sporulation and the improvement of nutrition synthesis in B150 allow itself to coexist with *K. vulgare* for a longer period in fermentation. Besides, the mutation of *comP* gene (encoding two-component sensor histidine kinase) improved the growth capability and extended the exponential phase^35^. The anti-stress protection was so indispensable for *K. vulgare* in fermentation. The mutation of NADPH nitroreductase in K150 and NADPH-dependent FMN reductase in B150 may enhance the ability of anti-oxidation. Particularity, the expression of dehydrogenases and the production of related acids increased significantly in K150.

Our laboratory has focused on the microbial consortia system for many years^36^,^37^,^38^,^39^,^40^. The complexity of the consortia system is that the isolation of the effect of each species alone will lack the interaction between two microorganisms, and vice versa. In the engineered microbial communities, the limitations imposed by metabolic load can be legitimately distributed for the members^41^. Many strategies have been used for the system optimization, such as genetic modification^39^,^42^, nutrient supply^43^,^44^ and fermentation process optimization^45^,^46^. The long-term adapted evolution in *K. vulgare*-*B. thuringiensis* consortium makes significant change for each other. It is hoped that our work will provide some ideas for the design and optimization of other microbial consortia systems.

## Material and Method

### Species and cultivation conditions

*B. thuringiensis* and *K. vulgare* were co-cultured into 250 mL flasks with 50 mL seed medium and shaking at 250 rpm, 30 °C, and transferred to fresh media every 24 hours^11^. The seed medium was composed of 2% L-sorbose, 0.3% corn-steep liquor (CSL), 1% peptone, 0.3% yeast extract, 0.3% beef extract, 0.1% urea, 0.1% KH_2_PO_4_, 0.02% MgSO_4_.7H_2_O and 0.1% CaCO_3_. After the 150^th^ serial subcultivation, samples from the co-cultures were purified on the solid agar plate. Samples of each purified *B. thuringiensis* (B0 and B150) and *K. vulgare* (K0 and K150) at exponential phase were obtained to extract the genomes and metabolites.

### Analyses of 2-KGA and biomass

The concentration of extracellular 2-KGA was determined by the High Performance Liquid Chromatography (HPLC) (Waters Corp., Massachusetts, USA), equipped with an Aminex HPX-87H column (Bio-Rad, CA) and a refractive index detector. The mobile phase used in the HPLC system was 5 mM H_2_SO_4_ at 65 °C with a flow rate of 0.6 mL/min. The cell density was measured as optical density at 600 nm (OD_600_) with a spectrophotometer after dissolving CaCO_3_ in 100 mM HCl.

### Genome sequencing and data processing

Isolation of genomic DNA was carried out using SDS method. Total DNA obtained was subjected to quality control by agarose gel electrophoresis and quantified by Qubit. The genomes were sequenced with MPS (massively parallel sequencing) Illumina technology. The DNA library was constructed as following: a paired-end library with an insert size of 350 bp. The 350 bp library was sequenced using an Illumina HiSeq 4000 by PE150 strategy. Library construction and sequencing was performed at the Beijing Novogene Bioinformatics Technology Co., Ltd. Quality control of paired-end reads were performed using in-house program. The original data obtained by high-throughput sequencing were transformed into raw sequenced reads by CASAVA base calling, and stored in FASTQ format, containing sequencing information and the corresponding sequencing quality information of the reads. The sequenced data were filtered and the sequence of adapter and low quality data were removed, resulting in the clean data used for subsequent analysis.

### The related nucleotide sequence accession numbers

The genome sequence of *K. vulgare* Hbe602 has been downloaded from GenBank via the accession numbers CP012908, CP012909 and CP012910. The genome sequence of *B. thuringiensis* Bc601 has been downloaded from GenBank via the accession numbers CP015150 (chromosome), CP015151 to CP015156 (six plasmids, respectively).

### Reads mapping and SNP/InDel/SV analysis

The variation information of the sample is obtained by aligning the reads with the designated reference. We mapped the reads to the reference sequence using BWA software^47^, counted the coverage of the reads to the reference sequence and made explanations of the alignment results using the SAMTOOLS software^48^. The variation map of the whole genome was created to show the distribution of SNP (single nucleotide polymorphism) and InDel informations. SNP mainly refers to the DNA sequence polymorphism caused by the single nucleotide variation at the genetic level, including transition and transversion. InDel refers to the insertion and deletion of small fragments in the genome. SAMTOOLS was used to the detection of the individual SNP/InDel in the functional regions of the genome. SV (structural variation) refers to the insertion, deletion, inversion and translocation of the large segments in the genome level. The insertion, deletion, inversion, intra-chromosomal translocation and inter translocation between the reference and the sample are found by BreakDancer software^49^.

### Metabolites extraction and derivatization

Cells cultured in different carbon source of logarithmic growth phase were quenched and extracted as intracellular metabolites according to our previous method^14^. An extra group of quenched cells was washed and dried to calculate the dry weight of the sampled cells. The 10 μL succinic *d*_4_ acid (0.1 mg/mL) was used as an internal standard to correct for minor variations occurring during sample preparation and analysis. The extracts of intracellular were lyophilized and four independent experiments were performed for each sample. Firstly, methoximation of the carbonyl groups was carried out by dissolving sample in 50 μL methoxamine hydrochloride (20 mg/mL in pyridine) and incubating it at 40 °C for 60 min. Then, 80 μL N-methyl-N-(trimethylsilyl) trifluoroacetamide (MSTFA) was added and it was incubated at 37 °C for 30 min for trimethylsilylation.

### Metabolomic analysis by GC-TOF/MS

Metabolites were analyzed by GC-TOF/MS (Waters Corp., USA). The 1 μL derivatized sample was injected by Agilent 7683 autosampler into GC (Agilent 6890) which was equipped with DB-5MS column (30 m × 0.25 mm × 0.25 μm, J&W Scientific, Folsom, CA). The oven temperature was programmed as 70 °C for 2 min, then increased to 290 °C (5 °C/min), holding for 3 min. The ion source temperature and ionization current were 250 °C and 40 μA, respectively. The mass scan range was 50–800 m/z. Peak detection, deconvolution and peak quantification were performed using Masslynx software 4.1. Metabolites were identified by comparing their mass fragmentation patterns with NIST mass spectral library^50^. The area of each acquired peak was normalized against the internal standard and dry cell weight. Multivariate data analysis was preformed by hierarchical cluster analysis (HCA) to view the relative differences in the metabolites concentrations among diverse conditions^51^.

## Additional Information

**How to cite this article:** Jia, N. *et al*. Comparative genomics and metabolomics analyses of the adaptation mechanism in *Ketogulonicigenium vulgare-Bacillus thuringiensis consortium. Sci. Rep.* **7**, 46759; doi: 10.1038/srep46759 (2017).

**Publisher's note:** Springer Nature remains neutral with regard to jurisdictional claims in published maps and institutional affiliations.

## Figures and Tables

**Table 1:** Validated mutations in K150/B150 of experimental evolution.

Species	Gene	Gene function	Mutation	Genome position/bp	Substitution
K150	*ffh*	signal recognition particle protein	C → T	201775	V → M
	KVH_05035	creatinine amidohydrolase	T → C	1003977	I → M
	*hipO*	amidohydrolase	G → A	1443879	G → S
	KVH_08735	NADPH nitroreductase	A → C	1743506	S → A
	*dksA*	RNA polymerase-binding protein DksA	G → A	2006178	R → C
B150	BtBc_02850	epimerase	C → T	550413	W → U
	BtBc_03145	chemotaxis protein	A → G	602604	Q → R
	*treR*	trehalose operon repressor	A → G	692130	Q → R
	*yfnA*	amino acid permease	C → T	917190	C → Y
	BtBc_04905	MarR family transcriptional regulator	G → A	932415	T → I
	BtBc_08920	hypothetical protein	C → T	1691716	M → I
	BtBc_08990	glycosyltransferase	A → G	1703700	T → A
	BtBc_12015	TetR family transcriptional regulator	A → G	2307900	M → T
	*azr*	NADPH-dependent FMN reductase	C → T	2370384	P → S
	*lsrB*	autoinducer 2-binding protein lsrB	T → A	2890716	S → T
	*hutH*	histidine ammonia-lyase	C → T	3779434	A → T
	*polC*	PolC-type DNA polymerase III	T → C	3942021	K → E
	*greA*	transcription elongation factor GreA	T → C	4475438	E → G
	*hemA*	glutamyl-tRNA reductase	T → C	4563120	H → R
	BtBc_24035	histone deacetylase	G → A	4743005	R → Q
	*mviM*	virulence factor MviM	A → G	4759919	Q → R
	*ftsK*	cell division protein FtsK	C → T	4773874	V → I
	BtBc_26105	serine/threonine protein kinase	G → A	5114831	A → V
	BtBc_27510	glycosyl transferase	C → T	5387774	W → U
	*rho*	transcription termination factor Rho	G → A	5448060	P → L
	BtBc_27985	LPXTG-domain-containing protein cell wall anchor domain	C → G	5485786	A → G
	BtBc_28330	glycosyl transferase family	T → C	5557300	S → P
	BtBc_30195	hypothetical protein	Delete T	1655	shift
	BtBc_30200	hypothetical protein	Insert A	2920	shift
	BtBc_08255	ribosomal protein L5 domain protein	Delete A	1568624	shift
	BtBc_08685	uracil-DNA glycosylase	Delete A	1648698	shift
	BtBc_09325	motility repressor MogR	Delete A	1775506	shift
	BtBc_12210	molecular chaperone Hsp20	Delete T	2343900	shift
	*bacF*	non-ribosomal peptide synthetase	Delete A	2446926	shift
	BtBc_16740	S-adenosylhomocysteine nucleosidase	Delete T	3298277	shift
	BtBc_18345	Lsa family ABC-F type ribosomal protection protein	Insert AT	3629982	shift
	BtBc_18600	spore germination protein	Insert A	3689846	shift
